# Lack of mutation in tumour-suppressor gene p53 in gestational trophoblastic tumours.

**DOI:** 10.1038/bjc.1996.233

**Published:** 1996-05

**Authors:** Y. F. Shi, X. Xie, C. L. Zhao, D. F. Ye, S. M. Lu, J. J. Hor, C. C. Pao

**Affiliations:** Women's Hospital, Zhejiang Medical University, Hangzhou, China.

## Abstract

**Images:**


British Journal of Cancer (1996) 73, 1216-1219
© 1996 Stockton Press All rights reserved 0007-0920/96 $12.00

### Lack of mutation in tumour-suppressor gene p53 in gestational trophoblastic tumours

Y-F Shi[1], X Xie[1], C-L Zhao[1], D-F Ye[1], S-M Lu[1], J Jen Hor[2] and CC Pao[2]

[1]*Women's Hospital, Zhejiang Medical University, Hangzhou, Zhejiang, China; and* [2]*Department of Biochemistry, Chang Gung College of Medicine and Technology, Taipei, Taiwan, China.*

**Summary** The objectives of this study were to better our understanding of the carcinogenesis of gestational trophoblastic tumours and to investigate the possible presence of mutational alteration of the p53 tumour-suppressor gene in these tumours. Amplification-based direct DNA sequencing was performed on 14 hydatidiform moles, six invasive moles, eight choriocarcinomas and ten normal early placental tissues. No mutation in exons 5-8 was detected in any of these 38 tissue specimens. These results suggest that a mutation in p53 tumour suppressor either does not exist or is a very rare event in gestational trophoblastic tumours. The gestational trophoblastic tumours probably involve a tumour-suppressor gene other than p53 gene or may follow a completely different pathway to their malignant phenotype.

**Keywords:** p53; tumour-suppressor gene; mutation; gestational trophoblastic tumour; polymerase chain reaction

Gestational trophoblastic diseases are a group of clinically and histopathologically defined entities with characteristics of reproductive failure and a high neoplastic potential (Roberts and Mutter, 1994). Major advances have been achieved during the past 40 years in the epidemiology, aetiology, pathology, endocrinology, immunology, diagnosis and treatment of gestational trophoblastic disease (Lurain, 1990). Early diagnosis and effective treatment of patients with gestational trophoblastic disease have resulted in almost 100% cure rates in non-metastatic disease and in the majority of patients with metastases (Lurain, 1990).

However, there is so far no reliable genetic marker for predicting which subset of moles will behave aggressively. For the most part, the pathogenesis and aetiology for hydatidiform mole and choriocarcinoma are still considered by many as controversial and unclear. Mutation of the p53 gene, which encodes a nuclear phosphoprotein of 393 amino acids, is the most common genetic alteration in human cancers (Greenblatt *et al.*, 1994). The p53 protein functions as tumour suppressor, which negatively regulates cell growth. Numerous recent reports have shown that missense or frameshift mutations of the p53 gene can be found in almost every type of tumour (Greenblatt *et al.*, 1994; Berchuck *et al.*, 1994) suggesting aberrations of possible common pathways of growth control in these diverse malignancies. Although mutations in the p53 gene have been identified throughout the gene, the vast majority (over 98%) of them locate in exons 5-8 regardless of the tumour type.

To evaluate the frequency of p53 mutation in the gestational trophoblastic tumours, we describe here the use of the polymerase chain reaction (PCR)-based direct DNA sequencing method to examine the DNA sequences of exons 5-8 of the p53 gene in 28 gestational trophoblastic tumours and in ten normal early placental tissues. Using this approach, no mutations were found in either the tumour or the normal placental tissues.

Correspondence: CC Pao, Department of Biochemistry, Chang Gung College of Medicine and Technology, 259 Wenhwa Road, Kweishan, Taoyuan, Taiwan, China
Received 21 August 1995; revised 9 November 1995; accepted 4 December 1995

### Materials and methods

### *Materials*

Serial 4-5 μm sections of paraffin-embedded tissues were prepared from 14 hydatidiform moles, six invasive moles, eight choriocarcinomas and ten normal early placental tissues (Table I). All tissues were taken from patients of the Women's Hospital, Zhejiang Medical University at Hangzhou and were obtained before any chemotherapy treatment. The following p53 mutant DNA specimens were obtained from Dr YS Chang of Chang Gung College of Medicine and Technology and served as mutant control DNA in the DNA sequencing experiments: laryngeal carcinoma no. 28, which contains a GTC to GCG (Val to Ala) mutation in codon 173 in exon 5; hypopharyngeal carcinoma no. 35, which contains a GTG to GTTG frameshift mutation on codon 203 in exon 6; nasopharyngeal carcinoma no. 81, which contains a CGG to CAG (Arg to Gln) mutation in codon 248 in exon 7; and laryngeal carcinoma no. 22, which contains an 8 bp deletion in codons 274-276 in exon 8 (Chang *et al.*, 1992).

### *Immunohistochemical staining of p53 protein in trophoblastic tissues*

Sections (4 μm) of formalin-fixed, paraffin-embedded tissues were cut. Sections were then deparaffinised, rehydrated and stained using the avidin-biotin-peroxidase complex method. Both anti-p53 antibody (clone DO-7) and conjugated secondary antibody (rabbit anti-mouse horseradish peroxidase) were obtained from Dako (Copenhagen). p53 staining results were scored by a modified version of Fromwitz's method with a combined tally for intensity of stained p53 signal and percentage of cells stained positive for p53 (Fromwitz *et al.*, 1987). Intensity of stained p53 signal was scored 3, 2, 1 and 0 representing strong, moderate, weak and negative signals respectively. The percentage of cells stained positive for p53 was also scored 3, 2, 1 and 0 representing >75%, 50-75%, 25-50% and <25% cells stained positive for p53 respectively. Therefore, the highest and lowest possible scores are 6 and 0 respectively. The Fromwitz scores were determined by C-L Z, who is a pathologist and had prior knowledge of the histopathological findings at the time of scoring.

**Table I** Fromwitz scores and histopathological findings of specimens

| Patient | Histology | Fromwitz score |
|---|---|---|
| 1 | Normal trophoblast | 0 |
| 2 | Normal trophoblast | 0 |
| 3 | Normal trophoblast | 0 |
| 4 | Normal trophoblast | 1 |
| 5 | Normal trophoblast | 0 |
| 6 | Normal trophoblast | 0 |
| 7 | Normal trophoblast | 0 |
| 8 | Normal trophoblast | 0 |
| 9 | Normal trophoblast | 0 |
| 10 | Normal trophoblast | 0 |
| 11 | Hydatidiform mole | 1 |
| 12 | Hydatidiform mole | 1 |
| 13 | Hydatidiform mole | 0 |
| 14 | Hydatidiform mole | 1 |
| 15 | Hydatidiform mole | 2 |
| 16 | Hydatidiform mole | 2 |
| 17 | Hydatidiform mole | 2 |
| 18 | Hydatidiform mole | 2 |
| 19 | Hydatidiform mole | 1 |
| 20 | Hydatidiform mole | 2 |
| 21 | Hydatidiform mole | 1 |
| 22 | Hydatidiform mole | 0 |
| 23 | Hydatidiform mole | 0 |
| 24 | Hydatidiform mole | 1 |
| 25 | Invasive mole | 1 |
| 26 | Invasive mole | 3 |
| 27 | Invasive mole | 4 |
| 28 | Invasive mole | 3 |
| 29 | Invasive mole | 3 |
| 30 | Invasive mole | 4 |
| 31 | Choriocarcinoma | 0 |
| 32 | Choriocarcinoma | 1 |
| 33 | Choriocarcinoma | 5 |
| 34 | Choriocarcinoma | 1 |
| 35 | Choriocarcinoma | 0 |
| 36 | Choriocarcinoma | 3 |
| 37 | Choriocarcinoma | 3 |
| 38 | Choriocarcinoma | 3 |

*DNA extraction and amplification of p53 gene DNA sequences by polymerase chain reaction*

Portions of each tissue section that contained cancer cells were identified under the microscope after haematoxylin and eosin staining and then removed by dissection for DNA extraction and subsequent analyses. Total cellular DNA was extracted from tissues by the standard phenol–chloroform method and purified by alcohol precipitation before being used for amplification of p53 gene DNA sequences. A total of five pairs of oligonucleotide primers based on published p53 gene DNA sequences were used to amplify exons 5, 6, 7 and 8 (exon 5 was amplified by two pairs of primers) (Soussi *et al.*, 1990). The sequence information for the primers is listed in Table II. The conditions used for the amplification reactions have been described earlier (Pao *et al.*, 1994) with minor modifications. Briefly, approximately 0.5 $\mu$g of purified cellular DNA was amplified with thermostable *Taq* DNA polymerase in a Thermal Cycler (Model 480, Perkin-Elmer Cetus, Norwalk, CT, USA). The 50 $\mu$l amplification reaction mixture contained 10 mmol $l^{-1}$ Tris-HCl, pH 8.3, 50 mmol $l^{-1}$ potassium chloride; 1.5 mmol $l^{-1}$ magnesium chloride; 0.01% gelatin; 10 pmole each of the primers for the initial amplification; 2.5 nmol each of the four deoxyribonucleoside triphosphates and 1 unit of *Taq* DNA polymerase (Perkin-Elmer Cetus). Amplification reactions were cycled 45 times beginning with 94°C for 30 s to denature the target DNA. This process was then followed by re-naturation for 30 s at either 55°C or 60°C, depending on the exons being amplified and primers used for most efficient amplification. All extension reactions were carried out at 72°C for 60 s. The amplified DNA products were confirmed by agarose gel electrophoresis. Because of the sensitivity of the PCR, a number of precautions were taken to minimise the possibility of contamination during sampling and subsequent processing (Pao *et al.*, 1991, 1993, 1994).

*DNA sequencing analysis*

Aliquots of 5 $\mu$l of each of the five sets of the amplification products were used to generate single-stranded DNA for subsequent direct sequencing of each exon of the p53 gene. Two asymmetric amplification reactions, one each with an excess of one of the primers over the other of opposite orientation, were performed in order to sequence from both directions. The single-stranded DNA was purified by repeated alcohol precipitation and washing. The resuspended DNA was then subjected to direct DNA sequencing using a Sequenase version 2.0 DNA Sequencing kit (United States Biochemical, Cleveland, OH, USA) according to the manufacturer's recommendation.

### Results

Immunohistochemical staining results indicated that p53 immunoreactivity ranged between 0 and 5 in the tissues examined (Table I and Figure 1). It appears that there is a statistically significant difference in the Fromwitz score among various histological types as determined by analysis of variance ($\chi^2 = 11.12$, $P < 0.001$). As a group, the invasive moles appear to have a higher score (mean = 3.0) than the other histological types. The hydatidiform mole group has a slightly higher profile (mean = 1.143) than the normal trophoblast group (mean = 0.1). The Fromwitz scores of the choriocarcinoma group are most variable with a mean of 2.0. Both modified t-test (least square difference or LSD method) and Duncan's multiple range test confirm that the Fromwitz

**Table II** Oligonucleotide primers used for p53 gene DNA amplification by polymerase chain reaction and primers used for direct DNA sequencing

| Target DNA | Primer sequences (from 5′ to 3′) | Size of amplified DNA (in base pairs) |
|---|---|---|
| Exon 5[a] | TTCCTCTTCCTGCAGTACTCCCCTGCCCTC<br>GTAGATGGCCATGGCGCGGACG | 129 |
| Exon 5[b] | GTTGATTCCACACCCCCGCCCGGCACCC<br>GCTCACCATCGCTATCTGAGC | 127 |
| Exon 6 | GATTGCTCTTAGGTCTGGCCCCTCCTCAGC<br>CAGACCTCAGGCGGCTCATAGG | 130 |
| Exon 7 | CTAGGTTGGCTCTGACTGTACCACCATCC<br>TGACCTGGAGTCTTCCAGTGTG | 118 |
| Exon 8 | GTAGTGGTAATCTACTGGGACGGAACAGC<br>CTCGCTTAGTGCTCCCTGGGGGC | 141 |

Exon 5 DNA of p53 gene was amplified and sequenced in two portions for greatest efficiency. [a]The upstream portion of the p53 gene. [b]The downstream portion of the p53 gene.

**Figure 1** Nuclear accumulation of p53 protein in choriocarcinoma. Strong p53 expression in the nuclei of choriocarcinoma cells from case number 33 as shown by immunohistochemistry (original magnifications ×470 in (**a**) and ×940 in (**b**).

**Figure 2** Agarose gel electrophoresis of PCR amplification products of p53 gene DNA sequences. Plasmid pGEM-3 DNA digested with a mixture of three restriction endonucleases (*Hin*fI, *Rsa*I and *Sin*I) was used as DNA size markers in the two outside lanes (lanes M), and the sizes of these DNA fragments are (from top to bottom) 2645, 1605, 1198, 676, 517, 460, 396, 350, 222, 179, 126, 75, 65, 51 and 36 bp. Lanes A and B are DNA from upstream and downstream portions of exon 5 of the p53 gene respectively. The amplified DNA are 129 and 127 base pairs respectively. Lanes C, D and E are DNA for exons 6, 7 and 8 respectively and their sizes are 130, 118 and 141 bp respectively.

scores are significantly associated with histological types, except between invasive moles and choriocarcinomas.

DNA of exons 5, 6, 7 and 8 of the p53 gene from a total of 38 tissue specimens (14 hydatidiform moles, six invasive moles, eight choriocarcinoma and ten normal placental tissues) were amplified and then sequenced successfully. The amplified DNA of p53 gene exon 5 (lanes A and B), 6 (lane C), 7 (lane D) and 8 (lane E) is illustrated in Figure 2. Exon 5 of the p53 gene was amplified and sequenced in two parts for greatest efficiency. The DNA sequencing results of the p53 gene confirmed that no mutation could be found in exons 5, 6, 7 and 8 of any of the 38 tissues studied (data not shown).

### Discussion

We have screened exons 5–8 of the p53 genes of 14 hydatidiform moles, six invasive moles, eight choriocarcinoma and ten normal early placental tissues by PCR amplification followed by direct DNA sequencing for the possible presence of genetic aberrations. Our research does not reveal any DNA sequence alteration in any of these 38 tissues. The regions of p53 genes we have examined include exons that are known for their functional importance (Ullrich *et al.*, 1992). The overwhelming majority (over 98%) of p53 gene mutations in tumour tissues and cancer cell lines reported so far in the literature are clustered within exons 5–8, equivalent to amino acid residues 130 and 290 of p53 protein (Hollstein *et al.*, 1991; Levine *et al.*, 1991). This is also a region where the DNA sequences are highly conserved among several different species (Soussi *et al.*, 1990; Pao *et al.*, 1994). In order to include any possible mutation that may occur at the intron–exon splicing junction, we chose to amplify the individual exon DNAs instead of amplifying the complete cDNA fragment in one single piece. It is, therefore, relatively safe to assume that we probably would have detected p53 mutations if they did exist.

There seems to be a trend between the Fromwitz score and the histological findings in the tissue specimens. However, p53 overexpression, as indicated by immunohistochemical staining, could be the result of actively proliferating cells and not necessarily due to the presence of a mutant form of p53 protein. This notion was supported by the observations of p53 overexpression in inflammatory lesions (Bosari *et al.*, 1993), and in the basal layers of warts and in the basal to middle third of cervical intraepithelial neoplasia lesions (Cooper *et al.*, 1993). Therefore, it would not be inconsistent to state that increased p53 expression correlates with histological type and that p53 mutations were not observed. Whether this is really the case would have to be examined further.

Genetic mutation in tumour-suppressor gene p53 is thought to contribute to tumour growth by inactivating proteins that normally act to limit cell proliferation (Ullrich *et al.*, 1992). A central role for p53 gene in transcription and phosphorylation events required for passage of a cell from $G_1$ to S-phase and in the decision of a cell to replicate or to go to apoptosis has become apparent (Levine *et al.*, 1994). Because of its central role in regulating cell growth and its potential association with the development of many cancer types, the structural integrity and expression of the p53 gene have been studied very extensively. p53 gene mutations are expected to be present in diverse malignancies and the vast majority of a large number of tumour types examined so far do contain mutations in the p53 gene. However, the mutational status of the p53 gene in gestational trophoblastic tumours has received relatively very little attention. Cheung *et al.* (1993) could not find any mutation in exons 5–8 of four hydatidiform moles after performing direct sequencing on amplified p53 cDNA fragments. On the other hand, Chen *et al.* (1994) were able to detect a sole missense point mutation in codon 295 of the p53 gene of a single hydatidiform mole patient among 24 patients examined. When these results and our data are taken together, it can be proposed that the p53 gene (or the mutant form of the p53 gene) is either not important or not directly involved in the oncogenesis of gestational trophoblastic tumours.

The complete lack of mutations in the p53 gene in cancers or in conditions related to cancer susceptibility are quite rare and have been reported only in paediatric astrocytomas (Litofsky *et al.*, 1994), malignant melanoma (Castresana *et al.*, 1993) and testicular cancer (Fleischhacker *et al.*, 1994). The reason for and significance of our failing to detect p53 genetic aberrations in gestational trophoblastic tumours are not completely clear at the present time. It is possible that some of these tumours contain mutations located outside the regions of p53, that we and others (Cheung *et al.*, 1993; Chen *et al.*, 1994) have examined. However, this prospect is not very likely because it would suggest involvement of regions of p53 proteins that are either not known to contain frequent mutations in cancers or to be considered functionally important. Another possibility is that there are other tumour-suppressor genes whose inactivation or loss of function is important in the carcinogenesis of these tumours, as has been suggested (Miyamoto *et al.*, 1991). Furthermore, it is also possible that carcinogenesis of gestational trophoblastic tumours, or at least certain subsets of them, may follow a completely different pathway to their malignant phenotype, such as telomere length and telomerase activity (Kim *et al.*, 1994; Wynford-Thomas *et al.*, 1995). The notion that most, if not all, gestational trophoblastic tumours contain only the wild-type p53 gene and that these tumours may arise through a transformation process other than genetic alteration of the p53 gene, is compatible with the fact that these tumours have an excellent (90–100%) chemotherapy cure rate. The link between the lack of a p53 mutation and the inherent high sensitivity to chemotherapy of gestational trophoblastic tumours is underscored by recent reports that loss of p53 function may reduce the response of malignant tumours to treatment (El Rouby *et al.*, 1993; Harris *et al.*, 1993).

In summary, the results of this study indicate that the gestational trophoblastic tumours either do not contain or very rarely contain genetic alterations in the functional important domains of the p53 tumour-suppressor gene. p53 gene may not play an important role in the carcinogenesis of these tumours. More studies are needed to better define the aetiology of gestational trophoblastic tumours.

**Acknowledgements**
This work was supported by research grant 39470723 from the National Natural Science Foundation of China awarded to YFS and by medical research grant CMRP-407 from Chang Gung College of Medicine and Technology and Memorial Hospital awarded to CCP. Authors gratefully acknowledge the assistance of Dr Sing Kai Lo in the statistical analysis of the data.

### References

BERCHUCK A, KOHLER MF, MARKS JR, WISEMAN R, BOYD J AND BAST RC Jr. (1994). The p53 tumour suppressor gene frequently is altered in gynecologic cancers. *Am. J. Obstet. Gynecol.*, **170**, 246–252.

BOSARI S, RONCALLI M, VIALE G, BOSSI P AND COGGI G. (1993). p53 immunoreactivity in inflammatory and neoplastic diseases of the uterine cervix. *J. Pathol.*, **169**, 425–430.

CASTRESANA JS, RUBIO MP, VAZQUEZ JJ, IDOATE M, SOBER AJ, SEIZINGER BR AND BARNHILL RL. (1993). Lack of allelic deletion and point mutation as mechanisms of p53 activation in human malignant melanoma. *Int. J. Cancer*, **55**, 562–565.

CHANG YS, LIN YJ, TSAI CN, SHU CH, TSAI MS, CHOO KB AND LIU ST. (1992). Detection of mutations in the p53 gene in human head and neck carcinomas by single strand conformation polymorphism analysis. *Cancer Lett.*, **67**, 167–174.

CHEN CA, CHEN YH, CHEN TM, KO TM, WU CC, LEE CN AND HSIEH CY. (1994). Infrequent mutation in tumour suppressor gene p53 in gestational trophoblastic neoplasia. *Carcinogenesis*, **15**, 2221–2223.

CHEUNG AN, SRIVASTAVA G, PITTALUGA S, MAN TK, NGAN H AND COLLINS RJ. (1993). Expression of c-myc and c-fms oncogenes in trophoblastic cells in hydatidiform mole and normal human placenta. *J. Clin. Pathol.*, **46**, 204–207.

COOPER K, HERRINGTON CS, EVANS MF, GATTER KC AND McGEE JO. (1993). p53 antigen in cervical condylomata, intraepithelial neoplasia and carcinoma: relationship to HPV infection and integration. *J. Pathol.*, **171**, 27–34.

EL ROUBY S, THOMAS A, COSTIN D, ROSENBERG CR, POTMESIL M, SILBER R AND NEWCOMB EW. (1993). p53 gene mutation in B-cell chronic lymphocytic leukemia is associated with drug resistance and is independent of MDR1/MDR3 gene expression. *Blood*, **82**, 3452–3459.

FLEISCHHACKER M, STROHMEYER T, IMAI Y, SLAMON DJ AND KEOFFLER HP. (1994). Mutations of the p53 gene are not detectable in human testicular tumors. *Mod. Pathol.*, **7**, 435–439.

FROMWITZ FB, VIOLA MV, CHAO S, ORAVEZ S, MISHRIKI Y, FINKEL G, GRIMSON R AND LUNDY J. (1987). Ras p21 expression in the progression of breast cancer. *Hum. Pathol.*, **18**, 1268–1275.

GREENBLATT MS, BENNETT WP, HOLLSTEIN M AND HARRIS CC. (1994). Mutations in the p53 tumour suppressor gene: clues to cancer etiology and molecular pathogenesis. *Cancer Res.*, **54**, 4855–4858.

HARRIS CC AND HOLLSTEIN M. (1993). Clinical implications of the p53 tumor-suppressor gene. *N. Engl. J. Med.*, **329**,1318–1327.

HOLLSTEIN M, SIDRANSKY D, VOGELSTEIN B AND HARRIS C. (1991). p53 mutations in human cancers. *Science*, **253**, 49–53.

KIM NW, PIATYSZEK MA, PROWSE KR, HARLEY CB, WEST MD, HO PL, COVIELLO GM, WRIGHT WE, WEINRICH SL AND SHAY JW. (1994). Specific association of human telomerase activity with immortal cells and cancer. *Science*, **266**, 2011–2015.

LEVINE AJ, MOMAND J AND FINLAY CA. (1991). The p53 tumor suppressor gene. *Nature*, **351**, 453–456.

LEVINE AJ, PERRY ME, CHANG A, SILVER A, DITTMER D, WU M AND WELSH D. (1994). The 1993 Walter Hubert Lecture: the role of the p53 tumour-suppressor gene in tumorigenesis. *Br. J. Cancer*, **69**, 409–416.

LITOFSKY NS, HINTON D AND RAFFEL C. (1994). The lack of a role for p53 in astrocytomas in pediatric patients. *Neurosurgery*, **34**, 967–972.

LURAIN JR. (1990). Gestational trophoblastic tumors. *Semin. Surg. Oncol.*, **6**, 347–353.

MIYAMOTO S, SASAKI M, NISHIDA M AND WAKE N. (1991). Identification of a chromosome carrying a putative tumor suppressor gene in human choriocarcinoma by microcell-mediated chromosome transfer. *Human Cell*, **4**, 38–43.

PAO CC, LIN SS, LIN CY, MAA JS, LAI CH AND HSIEH TT. (1991). Identification of human papillomavirus in peripheral blood mononuclear cells by DNA amplification method. *Am. J. Clin. Pathol.*, **95**, 540–546.

PAO CC, HOR JJ, TSAI PL AND HORNG MY. (1993). Inhibition of in vitro enzymatic DNA amplification reaction by ultraviolet light irradiation. *Mol. Cell. Probes*, **7**, 217–219.

PAO CC, KAO SM, CHEN JH, TANG GC, CHANG PY AND TAN TT. (1994). State of mutational alterations of the p53 and retinoblastoma susceptibility genes in papillomavirus-negative human small cell cervical carcinomas. *J. Surg. Oncol.*, **57**, 87–93.

ROBERTS DJ AND MUTTER GL. (1994). Advances in the molecular biology of gestational trophoblastic disease. *J. Reprod. Med.*, **39**, 201–208.

SOUSSI T, CARON DE FROMENTAL C AND MAY P. (1990). Structural aspects of the p53 protein in relation to gene revolution. *Oncogene*, **5**, 945–952.

ULLRICH SJ, ANDERSON CW, MERCER WE AND APPELLA E. (1992). The p53 tumor suppressor protein, a modulator of cell proliferation. *J. Biol. Chem..*, **267**, 15259–15262.

WYNFORD-THOMAS D, BOND JA, WYLLIE FS, and JONES CJ. (1995). Does telomere shortening drive selection for p53 mutation in human cancer? *Mol. Carcinogenesis*, **12**, 119–123.


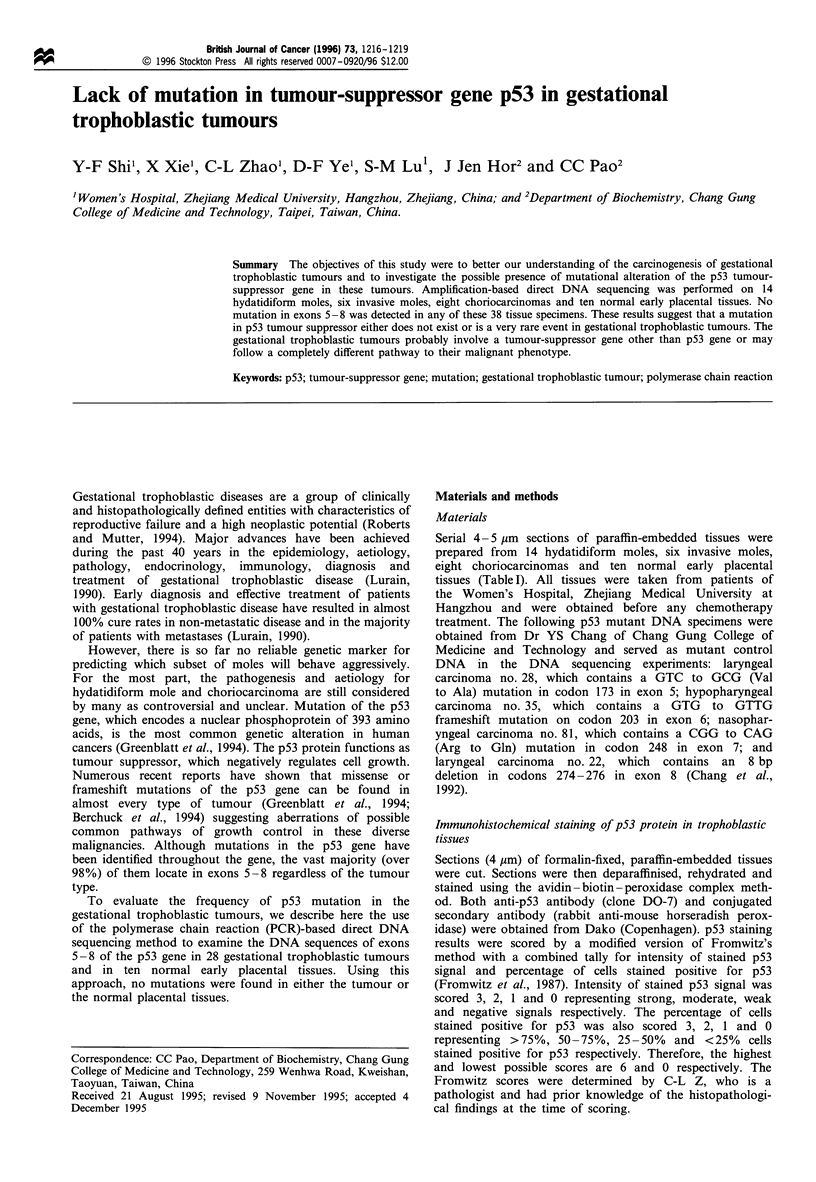

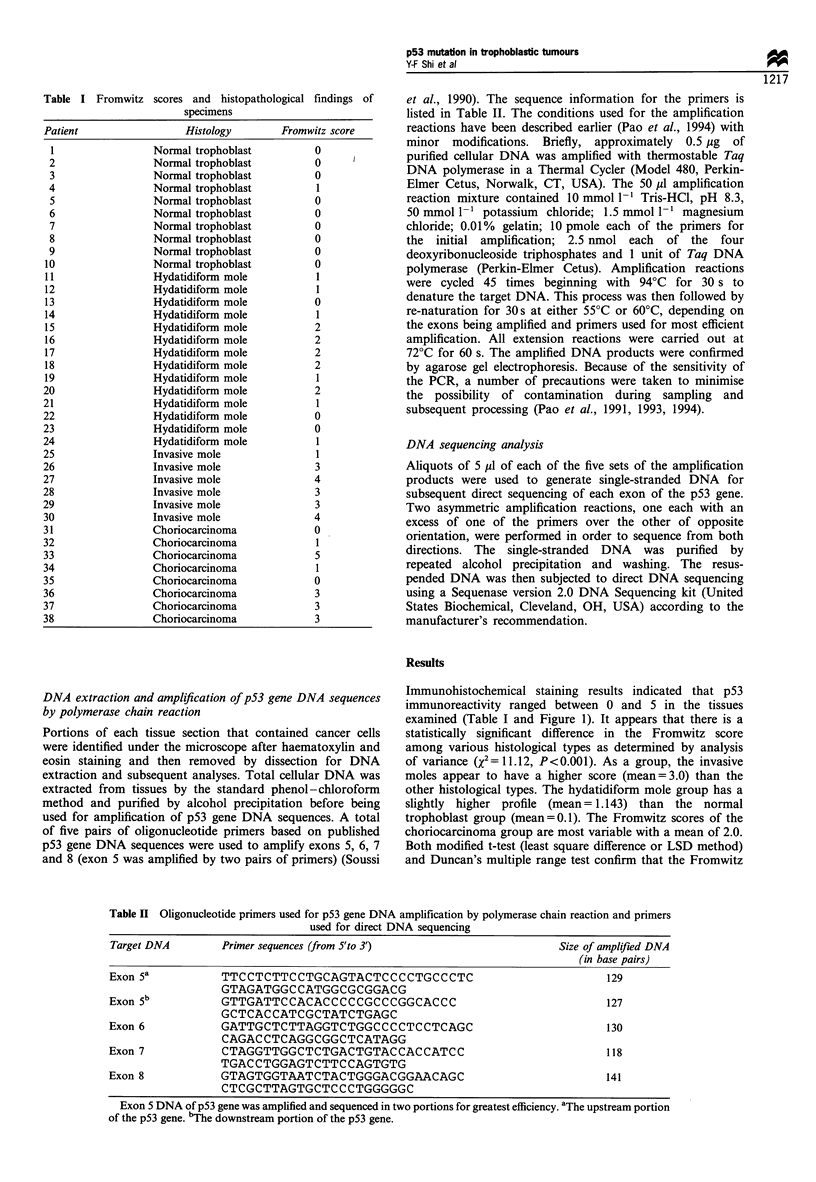

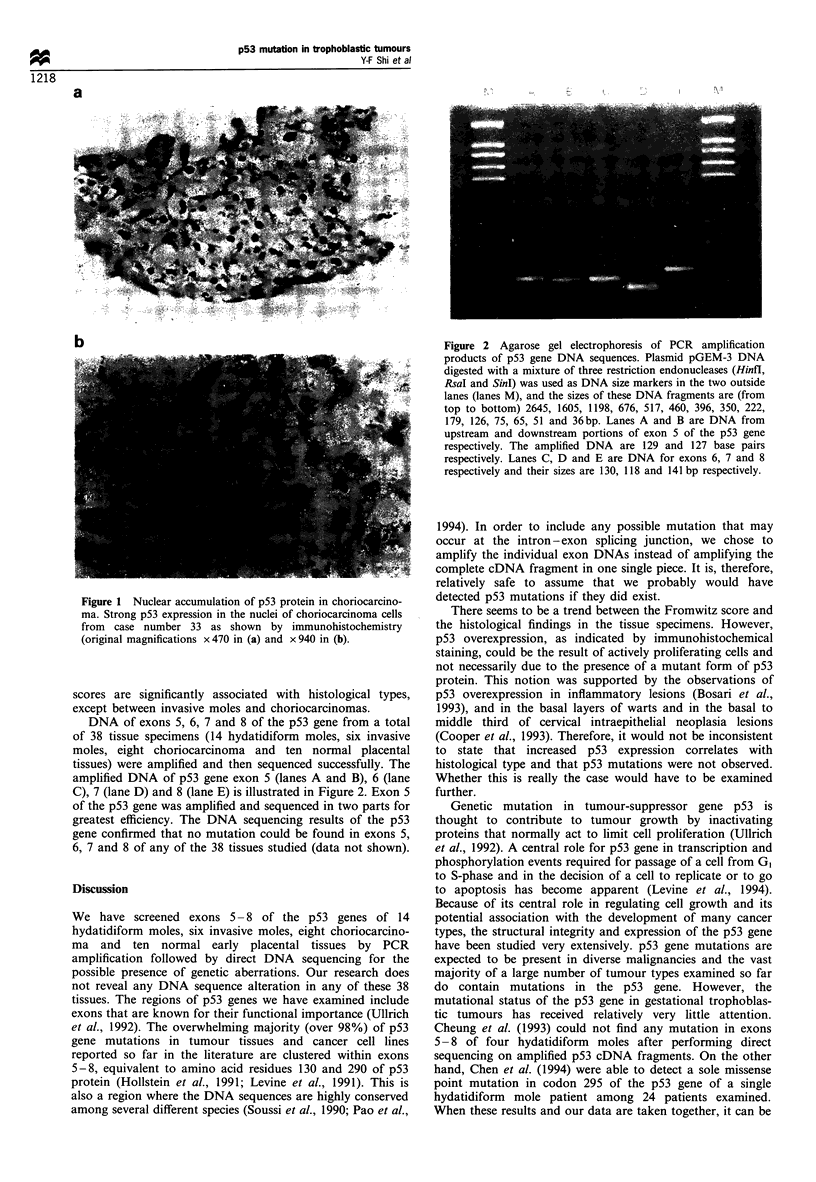

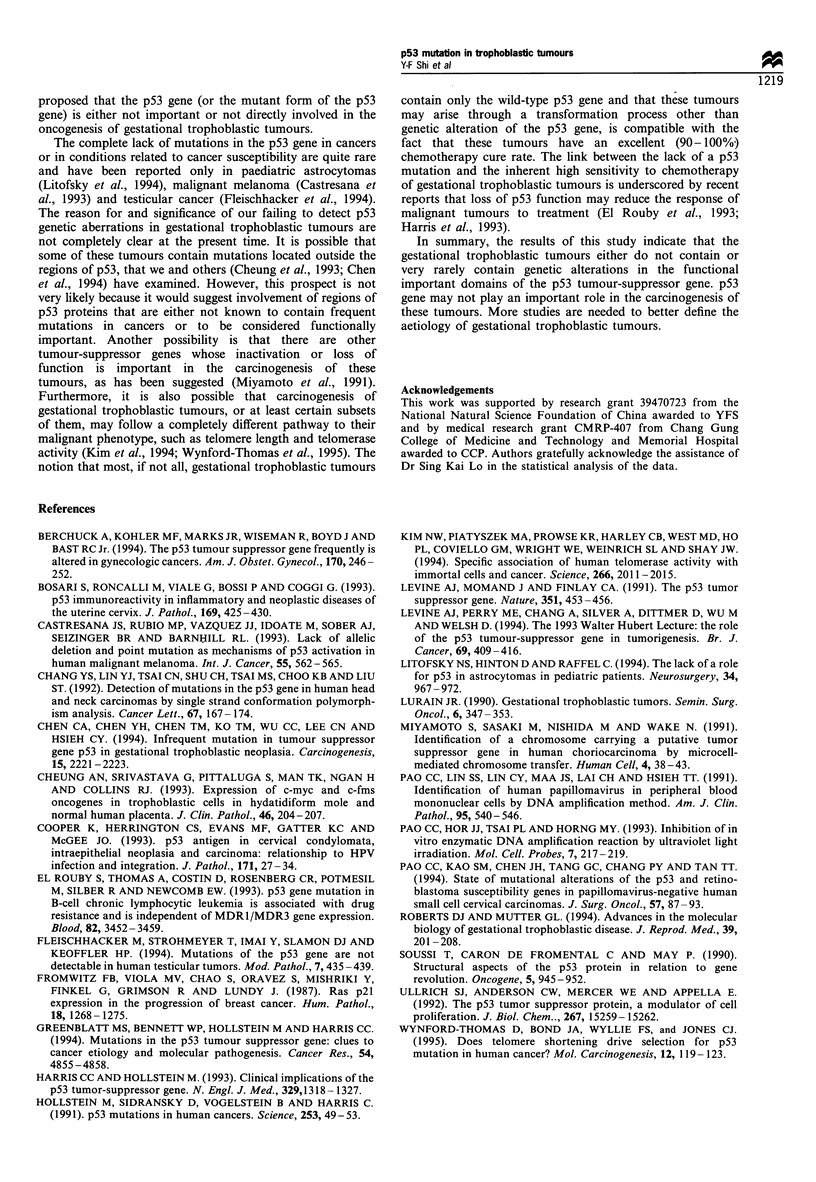


## References

[OCR_00525] Berchuck A., Kohler M. F., Marks J. R., Wiseman R., Boyd J., Bast R. C. (1994). The p53 tumor suppressor gene frequently is altered in gynecologic cancers.. Am J Obstet Gynecol.

[OCR_00530] Bosari S., Roncalli M., Viale G., Bossi P., Coggi G. (1993). p53 immunoreactivity in inflammatory and neoplastic diseases of the uterine cervix.. J Pathol.

[OCR_00535] Castresana J. S., Rubio M. P., Vázquez J. J., Idoate M., Sober A. J., Seizinger B. R., Barnhill R. L. (1993). Lack of allelic deletion and point mutation as mechanisms of p53 activation in human malignant melanoma.. Int J Cancer.

[OCR_00541] Chang Y. S., Lin Y. J., Tsai C. N., Shu C. H., Tsai M. S., Choo K. B., Liu S. T. (1992). Detection of mutations in the p53 gene in human head and neck carcinomas by single strand conformation polymorphism analysis.. Cancer Lett.

[OCR_00547] Chen C. A., Chen Y. H., Chen T. M., Ko T. M., Wu C. C., Lee C. N., Hsieh C. Y. (1994). Infrequent mutation in tumor suppressor gene p53 in gestational trophoblastic neoplasia.. Carcinogenesis.

[OCR_00554] Cheung A. N., Srivastava G., Pittaluga S., Man T. K., Ngan H., Collins R. J. (1993). Expression of c-myc and c-fms oncogenes in trophoblastic cells in hydatidiform mole and normal human placenta.. J Clin Pathol.

[OCR_00560] Cooper K., Herrington C. S., Evans M. F., Gatter K. C., McGee J. O. (1993). p53 antigen in cervical condylomata, intraepithelial neoplasia, and carcinoma: relationship to HPV infection and integration.. J Pathol.

[OCR_00570] Fleischhacker M., Strohmeyer T., Imai Y., Slamon D. J., Koeffler H. P. (1994). Mutations of the p53 gene are not detectable in human testicular tumors.. Mod Pathol.

[OCR_00574] Fromowitz F. B., Viola M. V., Chao S., Oravez S., Mishriki Y., Finkel G., Grimson R., Lundy J. (1987). ras p21 expression in the progression of breast cancer.. Hum Pathol.

[OCR_00580] Greenblatt M. S., Bennett W. P., Hollstein M., Harris C. C. (1994). Mutations in the p53 tumor suppressor gene: clues to cancer etiology and molecular pathogenesis.. Cancer Res.

[OCR_00586] Harris C. C., Hollstein M. (1993). Clinical implications of the p53 tumor-suppressor gene.. N Engl J Med.

[OCR_00590] Hollstein M., Sidransky D., Vogelstein B., Harris C. C. (1991). p53 mutations in human cancers.. Science.

[OCR_00594] Kim N. W., Piatyszek M. A., Prowse K. R., Harley C. B., West M. D., Ho P. L., Coviello G. M., Wright W. E., Weinrich S. L., Shay J. W. (1994). Specific association of human telomerase activity with immortal cells and cancer.. Science.

[OCR_00602] Levine A. J., Momand J., Finlay C. A. (1991). The p53 tumour suppressor gene.. Nature.

[OCR_00607] Levine A. J., Perry M. E., Chang A., Silver A., Dittmer D., Wu M., Welsh D. (1994). The 1993 Walter Hubert Lecture: the role of the p53 tumour-suppressor gene in tumorigenesis.. Br J Cancer.

[OCR_00612] Litofsky N. S., Hinton D., Raffel C. (1994). The lack of a role for p53 in astrocytomas in pediatric patients.. Neurosurgery.

[OCR_00615] Lurain J. R. (1990). Gestational trophoblastic tumors.. Semin Surg Oncol.

[OCR_00621] Miyamoto S., Sasaki M., Nishida M., Wake N. (1991). [Identification of a chromosome carrying a putative tumor suppressor gene in human choriocarcinoma by microcell-mediated chromosome transfer].. Hum Cell.

[OCR_00631] Pao C. C., Hor J. J., Tsai P. L., Horng M. Y. (1993). Inhibition of in vitro enzymatic DNA amplification reaction by ultra-violet light irradiation.. Mol Cell Probes.

[OCR_00636] Pao C. C., Kao S. M., Chen J. H., Tang G. C., Chang P. Y., Tan T. T. (1994). State of mutational alterations of p53 and retinoblastoma susceptibility genes in papillomavirus-negative small cell cervical carcinomas.. J Surg Oncol.

[OCR_00627] Pao C. C., Lin S. S., Lin C. Y., Maa J. S., Lai C. H., Hsieh T. T. (1991). Identification of human papillomavirus DNA sequences in peripheral blood mononuclear cells.. Am J Clin Pathol.

[OCR_00644] Roberts D. J., Mutter G. L. (1994). Advances in the molecular biology of gestational trophoblastic disease.. J Reprod Med.

[OCR_00647] Soussi T., Caron de Fromentel C., May P. (1990). Structural aspects of the p53 protein in relation to gene evolution.. Oncogene.

[OCR_00652] Ullrich S. J., Anderson C. W., Mercer W. E., Appella E. (1992). The p53 tumor suppressor protein, a modulator of cell proliferation.. J Biol Chem.

[OCR_00657] Wynford-Thomas D., Bond J. A., Wyllie F. S., Jones C. J. (1995). Does telomere shortening drive selection for p53 mutation in human cancer?. Mol Carcinog.

[OCR_00566] el Rouby S., Thomas A., Costin D., Rosenberg C. R., Potmesil M., Silber R., Newcomb E. W. (1993). p53 gene mutation in B-cell chronic lymphocytic leukemia is associated with drug resistance and is independent of MDR1/MDR3 gene expression.. Blood.

